# High protein diet maintains glucose production during exercise-induced energy deficit: a controlled trial

**DOI:** 10.1186/1743-7075-8-26

**Published:** 2011-04-28

**Authors:** Tracey J Smith, Jean-Marc Schwarz, Scott J Montain, Jennifer Rood, Matthew A Pikosky, Carmen Castaneda-Sceppa, Ellen Glickman, Andrew J Young

**Affiliations:** 1 U.S. Army Research Institute of Environmental Medicine, Military Nutrition Division, Kansas Street, Building 42, Natick, MA 01760, USA; 2 Pennington Biomedical Research Center, 6400 Perkins Road, Baton Rouge, LA 70808, USA; 3 Touro University, Department of Osteopathic Medicine, 1310 Johnson Lane, Mare Island, Vallejo, CA 94592, USA; 4 Jean Mayer USDA Human Nutrition Research Center on Aging at Tufts University, 711 Washington Street, Boston, MA 02111-1524, USA

**Keywords:** carbohydrate metabolism, gluconeogenesis, protein intake, negative energy balance, stable isotopes

## Abstract

**Background:**

Inadequate energy intake induces changes in endogenous glucose production (GP) to preserve muscle mass. Whether addition provision of dietary protein modulates GP response to energy deficit is unclear. The objective was to determine whether exercise-induced energy deficit effects on glucose metabolism are mitigated by increased dietary protein.

**Methods:**

Nineteen men ([mean ± SD] 23 ± 2 y, VO_2peak _59 ± 5 ml·kg^-1^·min^-1^) were divided into three groups, two consuming moderate (MP; 0.9 g protein kg^-1 ^d^-1^), and one high (HP; 1.8 g protein kg^-1 ^d^-1^) protein diets (55% energy from carbohydrate) for 11 days. Following 4 days of energy balance (D1-4), energy expenditure was increased for 7 days (D5-12) in all groups. Energy intake was unchanged in two, creating a 1000 kcal d^-1 ^deficit (DEF-MP, DEF-HP; n = 6, both groups), whereas energy balance was maintained in the third (BAL-MP, n = 7). Biochemical markers of substrate metabolism were measured during fasting rest on D4 and D12, as were GP and contribution of gluconeogenesis to endogenous glucose production (*f*_gng_) using 4-h primed, continuous infusions of [6,6-^2^H_2_]glucose (dilution-method) and [2-^13^C]glycerol (MIDA technique). Glycogen breakdown (GB) was derived from GP and *f*_gng_.

**Results:**

Plasma β-hydroxybutyrate levels increased, and plasma glucose and insulin declined from D4 to D12, regardless of group. DEF-MP experienced decreased plasma GP from D4 to D12 ([mean change ± SD] 0.24 ± 0.24 mg·kg^-1^·min^-1^), due to reduced GB from D4 (1.40 ± 0.28 mg·kg^-1^·min^-1^) to D12 (1.16 ± 0.17 mg·kg^-1^·min^-1^), P < 0.05. Conversely, BAL-MP and DEF-HP sustained GP from D4 to D12 ([mean change ± SD] 0.1 ± 0.5 and 0.0 ± 0.2 mg·kg^-1^·min^-1^, respectively) by maintaining GB.

**Conclusion:**

Exercise-induced energy deficit decreased GP and additional dietary protein mitigated that effect.

## Background

Energy deficit induced by energy restriction or by the accumulation of additional physical activity results in negative nitrogen and protein balance [1,2]. Short-term fasting leads to increased leucine flux, leucine oxidation, and accelerated proteolysis, and multiple weeks of energy-restriction is commonly accompanied by reductions in lean mass [3-6]. As a consequence, there has been interest in whether dietary protein supplementation during periods of energy restriction might be advantageous. We recently observed that dietary protein supplementation, when energy expenditure was abruptly increased 1,000 kcal per day (for seven days), attenuated the negative nitrogen balance accompanying energy deficit [7], whereas others have reported the dietary protein supplementation assists in preservation of lean mass during periods of persistent energy deficit [8-10].

A separate consequence of energy deficit is a reduction in hepatic glucose production [11,12]. When in energy balance, and fluxes through intrahepatic glucose-producing metabolic pathways are measured at rest, gluconeogenesis contributes approximately one-third and glycogenolysis contributes approximately two-thirds to the glucose released into the blood. Prolonged fasting (60 h) leads to hepatic glucose down-regulation and glucose appearance shifts almost entirely to gluconeogenesis due to the depletion of hepatic glycogen stores [11,13]. Whether provision of gluconeogenic precursors, via provision of amino acids, affects the down regulation of hepatic glucose production has not been examined.

The primary aim of the present investigation was to determine if the rate of endogenous glucose production declines in response to energy deficit produced by an abrupt increase in energy expenditure and to ascertain if dietary manipulation-specifically, provision of additional dietary protein with concomitant negative energy balance-could attenuate this effect. The working hypothesis was that provision of gluconeogenic precursors via additional dietary protein would attenuate the fall in glucose production accompanying energy deficit without a concomitant increase in energy intake. Further, this study sought to determine the contribution of gluconeogenesis and glycogenolysis, and biomarkers of substrate metabolism, to observed changes in endogenous glucose production to the exercise and diet manipulations. This experiment was part of a broad study assessing the adequacy of current recommendations regarding optimal dietary protein content for persons such as athletes or deployed military personnel who experience periods of high energy expenditures without an adequate increase in energy intakes [7].

## Methods

This study was approved by institutional review boards at the U.S. Army Research Center of Environmental Medicine and at Tufts University. Twenty-six healthy men gave their free informed, voluntary, written consent to participate in this investigation following an oral and written explanation of the study procedures and risks. All subjects completed an initial screening form and were medically cleared for participation. Subjects were required to be weight stable (±2.2 kg) for two months prior to the start of the study, have a 6 month aerobic training history (≥ 5 d·wk^-1^, 30 min·d^-1^), and a VO_2peak _± 54 or 52 ml^.^kg^-1.^min^-1 ^for ages 18-29 and 30-35 y, respectively. VO_2peak _guidelines were established to represent the fitness characteristics of infantry Soldiers and competitive athletes. Individuals who used tobacco products, had a disease or took medication known to impact macronutrient metabolism, or possessed any cardiovascular or musculoskeletal conditions that prohibited strenuous exercise, were excluded. The investigators adhered to the policies for protection of human subjects as prescribed in Army Regulation 70-25 and USAMRMC Regulation 70-25, and the research was conducted in adherence with the provisions of 32 CFR Part 219.

### Experimental Design

**Figure 1:**
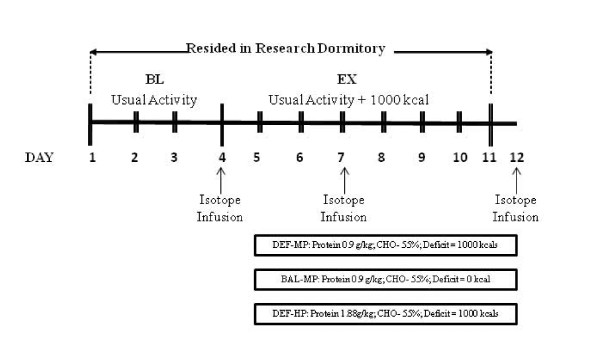
**Experimental Design**. This figure presents the experimental design. During the first four days of the experimental phase (D1-4), all groups were in energy balance, with two groups consuming 0.9 g protein·kg body weight^-1^·d^-1 ^and the third group consuming 1.8 g protein·kg body weight^-1^·d^-1^. Beginning on day 5, all volunteers increased their energy expenditure by 1,000 kcals·d^-1 ^via exercise. In one group assigned to the diet containing 0.9 g protein·kg body weight^-1^·d^-1^, the increased energy expenditure was accompanied by concomitant increase in energy intake to maintain energy balance during the intervention period (D5-11) (BAL-MP). In the other two groups, who were assigned to diets containing 0.9 g protein·kg body weight^-1^·d^-1 ^(DEF-MP) or 1.8 g protein·kg body weight^-1^·d^-1 ^(DEF-HP), energy intake remained unchanged.

Volunteers resided in a research dormitory for the duration of the experimental phase of the study allowing for strict control and monitoring of energy intake and energy expenditure (Figure 1). During the first four days of the experimental phase (D1-4), all groups were in energy balance, with two groups assigned to a diet containing 0.9 g protein·kg body weight^-1^·d^-1 ^and the third group assigned to a diet containing 1.8 g protein·kg body weight^-1^·d^-1^. The lower protein level was selected as Meredith et al. (1989) had previously determined that 0.94 g protein per kg body weight was necessary to achieve nitrogen balance in men expending 3,500-4,200 kcals/d weight [14]. Energy intake and expenditure was individually matched to subjects' normally accustomed levels. The normal accustomed level was assessed by diet and physical activity records subjects maintained for 3 days before beginning the experimental phase of the study. Beginning on day 5, all volunteers increased their energy expenditure by 1,000 kcals·d^-1 ^by exercising at intensities corresponding to 50-65% VO_2peak_. In one group, assigned to the diet containing 0.9 g protein·kg body weight^-1^·d^-1^, increased energy expenditure was accompanied by concomitant increase in energy intake to maintain energy balance during the intervention period (D5-11) (BAL-MP). In the other two groups, assigned to diets containing either 0.9 g protein·kg body weight^-1^·d^-1 ^(DEF-MP) or 1.8 g protein·kg body weight^-1^·d^-1 ^(DEF-HP), energy intake remained unchanged.

Baseline testing included assessment of aerobic capacity (peak oxygen uptake [VO_2peak_]), anthropometry (height and weight), body composition (dual-energy x-ray absorptiometry or DEXA), 3-day diet records, and 3-day physical activity records. Descriptions of these measures are given below.

### Peak Oxygen Uptake

Peak oxygen uptake (VO_2peak_) was determined by analysis of expired gases over the final minute of a cycle ergometer test using indirect open circuit spirometry (True Max 2400, Parvomedics, Sandy, Utah). The protocol was progressive in intensity, continuous in nature and performed indoors at a temperature of 20-22°C and 30-80% humidity. Volunteers first pedaled for 3 min at 100W, and intensity was increased by 30W every 2 min until the volunteer was unable to maintain a pedaling rate that maintained or increased O_2 _consumption.

### Anthropometry

Vertical height was measured in duplicate to the nearest 0.1 cm. Subjects were measured in stocking feet and standing on a flat surface, feet together, knees straight, and the head, shoulder blades, buttocks, and heels in contact with a vertical wall. Body weight was measured at baseline and twice daily (prior to morning meal, and following the evening meal) using a calibrated electronic battery-powered scale accurate to 0.1 kg. Body composition was determined by DEXA at baseline and D11.

### Baseline Dietary and Physical Activity Records

Three-day dietary and physical activity records were collected from each volunteer before the experimental phase of the study began to assess usual nutrient intake for energy, carbohydrates, protein, and fat and usual energy expenditure. All dietary records were analyzed by computer-based nutrient analysis software, Food Processor version 8.5.0 (ESHA Research, Salem, OR). Each subject's normal daily activities were grouped into different categories by duration and corresponding metabolic equivalent (MET) and average daily energy expenditure determined. The average daily energy intake determined from these records was used in combination with the average daily energy expenditure determined from the physical activity records to establish the energy intake and expenditure levels for the first four days of the experimental phase of the study (D1-4).

### Dietary Intervention

Three to five days prior to starting the study, subjects received instruction on a diet that contained the same amount of protein as the study diet. During the controlled study, diet consisted of whole foods and liquid supplements. Portions of all foods and liquids were weighed by a Registered Dietitian. A dairy-based liquid meal replacement (Boost^® ^Plus, NestlèNutrition, Vevey, Switzerland) was the main dietary protein source, and was consistent across groups. Protein content was held constant throughout the study for all groups. Total energy intake for study days 1-4 was individualized to match each volunteer's usual energy intake and expenditure in order to maintain energy balance. DEF-MP and DEF-HP maintained the same energy intake during the period of increased energy expenditure as during the baseline period (days 1-4), while energy intake was increased for BAL-MP to match the increase in energy expenditure. Total energy content was adjusted by adding or subtracting fat and carbohydrate containing foods so that the caloric ratio of these nutrients in the diet remained approximately 1:2, respectively. Meals were served according to a fixed schedule, but water and sugar-free non-caffeinated drinks were allowed ad libitum.

### Exercise Intervention

Usual/Normal Exercise Period: During study days 1-4, each subject's normal daily activities were grouped into different categories by duration and corresponding metabolic equivalent (MET) level. The type, intensity, and duration of all activities were tightly controlled by dividing each 24 hour period into prescribed 15 minute blocks at a specific MET level in order to duplicate the subjects' normal daily caloric expenditure.

### Increased exercise period

During study days 5-11, volunteers increased their energy expenditure by 1,000 kcal each day by exercising between 50-65% of their VO_2peak _for approximately 80 minutes. For subject comfort and to avoid injuries, exercise was divided between various modalities (bike, treadmill, elliptical) and split into 15 min intervals throughout the day. Volunteers were allowed to self-select how they wanted to distribute these 15 minute blocks as long as they completed the prescribed daily activity energy expenditure.

### Determination of Total Daily Energy Expenditure (TDEE)

Energy expenditure was determined periodically throughout the day using indirect open circuit spirometry (True Max 2400, Parvomedics, Sandy, Utah). TDEE was calculated using the following equation:

EE_sleep _was estimated via measurement of basal metabolic rate on days 1, 2, 5, and 8, EE_physical activity _was measured during each prescribed exercise activity on days 1, 5, and 8, and EE_miscellaneous _was measured during non-exercise activities (i.e. watching TV, playing video games, reading) on days 1, 5, and 8.

### Isotope-Infusion Protocol

Endogenous rate of glucose production and the contribution of gluconeogenesis to endogenous glucose production was assessed in the fasted state at rest on D4 and D12, using a priming dose of [6,6-^2^H_2_]glucose and [^13^C]glycerol and 4-h primed, continuous infusions of [6,6-^2^H_2_]glucose and [^13^C]glycerol. Subjects began fasting at 1800 hours on the evening before each isotope-infusion protocol, and were transported to the Jean Mayer USDA Human Nutrition Research Center on Aging at Tufts University (Boston, MA) where they remained overnight in the Metabolic Research Unit under the supervision of nursing staff. Subjects were awakened the following morning at ~0450 hours. A venous catheter was inserted into an antecubital vein for isotope infusion. Another catheter was placed in the contralateral hand vein, and the hand was heated with a heating pad, for the sampling of "arterialized" blood [15]. At 0600 hours, subjects were infused with a priming dose of [6,6 ^2^H_2_]glucose (26.4 μmol·kg^-1^) and [2-^13^C]glycerol (190 μmol·kg^-1^), followed by a 4-h continuous infusion (50 ml·h^-1^) of [6,6 ^2^H_2_]glucose (19.8 μmol·kg^-1^·h^-1^) and [2-^13^C]glycerol (189 μmol·kg^-1^·h^-1^). "Arterialized" venous blood samples were drawn immediately before the priming doses were administered, at 60-min intervals during the first two hours of the infusion, and at 15-min intervals during the final two hours of the infusion for subsequent analyses of plasma isotope enrichment. Resting VO_2 _and CO_2 _production rate (i.e., respiratory exchange ratio) were measured before the priming infusions, and for the last five minutes of each hour of the infusion. At the completion of the infusion period, catheters were removed, the subjects' arms bandaged and the volunteers were transported back to USARIEM to resume diet and exercise regimes as appropriate.

### Calculations

Rates of endogenous glucose appearance were calculated using equations described by Wolfe et al. [16] after correction for the doubly labeled glucose generated by gluconeogenesis, when two labeled 13C trioses are combined to form glucose [11]. The plasma rates of appearance (R_a_) of unlabelled glucose were obtained from dilution of infused glucose by the following equation:

where E_i _is the enrichment of the infused glucose tracer, E_p _is the enrichment of the glucose tracer in the blood plasma at a steady state, and i is the infusion rate of the glucose tracer. Steady state was achieved by ensuring that the plateau steady state plasma enrichment of [6,6-^2^H_2_]glucose measurements did not fluctuate more than 5% from the mean. Plateau values exceeding 5% variation from the mean were rerun on the gas chromatography-mass spectroscope. Rates of endogenous glucose appearance (endogenous glucose production or GP) are then derived from the following equation:

where exogenous glucose includes the tracer and infused glucose (i.e. priming dose).

Contribution of gluconeogenesis to endogenous glucose production (*f*_gng_) was determined from mass isotopomer distribution analysis on plasma glucose from [2-^13^C]glycerol, and calculated using the equations described by Hellerstein et al. [11]. Absolute flux from glycogen to plasma glucose [glycogen breakdown (GB)], was calculated by difference from *f*_gng_.

### Other Biochemical Analyses

Fasting blood samples, which were drawn on isotope infusion protocol days (D4 and D12), were analyzed for glucose, glycerol, free fatty acids, β-Hydroxybutyrate, glucagon, and insulin. All analyses were completed by the Pennington Biomedical Research Center (Baton Rouge, LA). Glucose was analyzed on a Synchron CX7 (Beckman-Coulter, Brea, CA) using a glucose oxidase electrode. β-Hydroxybutyrate was measured on a Synchron CX5 (Beckman-Coulter, Brea, CA) using reagents from Sigma (St Louis, MO). Insulin was determined using immunoassays with fluorescence detection on an Immulite 2000 instrument (Diagnostic Products Corporation, Los Angeles, CA). Free fatty acids and glycerol were analyzed on a Synchron CX5 using enzymatic reaction with colorimetric detection. Lastly, glucagon was measured by radioimmunoassay on a Packard Riastar gamma counter (Diagnostic Products Corporation, Los Angeles, CA).

### Statistical Analysis

Results are presented as means (± SD). Statistical analysis was completed using the SPSS statistical package version 15.0 (SPSS Inc., Chicago, IL). Student's T-test was utilized to compare pre- and post-study anthropometric and body composition data (i.e. body weight, body fat, and fat-free mass). Of principal interest in this study was the effect of diet (DEF-MP vs. BAL-MP vs. DEF-HP) on GP, *f*_gng_, and GB. Data from Christiansen et al. [17] was used to determine sample size. Five subjects per group are necessary to detect a 0.44 g·kg body weight^-1^·min^-1 ^difference in endogenous glucose production between groups (from D4 to D12) with a standard deviation of 0.20 g·kg body weight^-1^_·_min^-1^, alpha equal to 0.05 and power at 0.8. The potential for time to affect GP, *f*_gng_, GB, respiratory exchange ratio (RER), substrate oxidation rates, and blood chemistries (i.e. glucose, glycerol, free fatty acids, β-Hydroxybutyrate, glucagon, and insulin) was assessed using a two-way (diet × time) repeated measures analysis of variance including D4 and D12. If significant main or interaction effects were observed, Bonferroni's correction was employed to identify differences among means. The impact of diet on GP, *f*_gng_, GB, and blood chemistries were also examined by comparing the mean and 95% confidence intervals (CI) for group differences (D12 - D4) similar to equivalence testing [18]. This procedure is a corollary for significance testing and provides insight into the importance of the effect magnitude [19]. Statistical comparisons of these mean changes (D12 - D4) between diet manipulations (DEF-MP, BAL-MP and DEF-HP) were made using a one-way analysis of variance. A "p" value of ≤ 0.05 was considered statistically significant.

## Results

### Subject Characteristics

Complete isotope infusion data was collected on 19 of the 26 recruited volunteers due to study withdrawal (e.g., illness or injury) or incomplete blood samples during the isotope infusion tests. Descriptive characteristics of the volunteers in the final sample (n = 19) are presented in Table 1. Baseline weight (kg) and FFM (kg) was significantly higher for DEF-HP compared with BAL-MP and DEF-MP. Groups were matched according to VO_2peak _(ml·kg^-1^·min^-1^).

**Table 1:** Baseline Subject Characteristics

	BAL-MP (n = 6)	DEF-MP (n = 7)	DEF-HP (n = 6)
Age (years)	24 ± 2	23 ± 2	23 ± 2
Height (cm)	177 ± 6	179 ± 3	182 ± 3
Weight (kg)	76 ± 3	73 ± 3	85 ± 4
Body Fat (%)	16 ± 3	13 ± 3	14 ± 2
Fat-free mass (kg)	64 ± 5	63 ± 5	73 ± 8
VO^2peak ^(ml·kg^-1^·min^-1^)	57 ± 8	59 ± 4	62 ± 4

Values expressed as means ± SD

### Dietary Intervention

Actual dietary intakes for BAL-MP, DEF-MP and DEF-HP are presented in Table 2. The diet provided approximately 8-12.6% protein, 32-37% fat, and 55% carbohydrate. Protein intake in BAL-MP and DEF-MP was slightly higher than prescribed (~1.0 g·kg^-1^·d^-1^), while the DEF-HP met the prescribed level during BL and EX (~1.8 g·kg^-1^·d^-1^). Mean energy deficits (kcal·d^-1^) for study days 5-11 were -33 ± 175, 868 ± 176, and 1001 ± 92 for BAL-MP, DEF-MP and DEF-HP, respectively. There was no difference in energy deficit between DEF-MP and DEF-HP.

**Table 2:** Actual Dietary Intake

	BAL-MP		DEF-MP		DEF-HP	
	**Baseline (D1-4)**	**Intervention (D5-11)**	**Baseline (D1-4)**	**Intervention (D5-11)**	**Baseline (D1-4)**	**Intervention (D5-11)**

Energy Intake (kcals·d^-1^)	3767 ± 700	4809 ± 612	3620 ± 404	3578 ± 438	3810 ± 1048	3807 ± 1048
Protein (g·kg^-1^·d^-1^)	1.0 ± 0.0	1.0 ± 0.0	1.0 ± 0.0	1.0 ± 0.0	1.7 ± 0.0	1.7 ± 0.0
Protein (% total intake)	8 ± 1	6 ± 0.7	8 ± 1	8 ± 1	15 ± 2	15 ± 2
Carbohydrates (% total intake)	54 ± 1	55 ± 1	56 ± 2	56 ± 2	56 ± 2	56 ± 2
Fat (% total intake)	38 ± 2	39 ± 2	36 ± 1	36 ± 1	29 ± 3	29 ± 3

Values expressed as means ± SD

### Body Composition

DEF-MP and DEF-HP experienced significant decreases in body weight (-2.4 ± 0.6 kg and -2.9 ± 1.0 kg, respectively), body fat (-0.9 ± 1.1% and -1.3 ± 0.9, respectively), and fat free mass (-1.6 ± 1.0 kg and -1.4 ± 0.9 kg, respectively) from D4 to D12. There were no significant differences in body weight, body fat or fat free mass for BAL-MP. There were no differences between groups for these outcome measures.

### Respiratory Exchange Ratios, VO_2 _and Substrate Oxidation Rate

Respiratory exchange ratios (RER), VO_2_, and substrate oxidation rates are presented in Table 3. VO_2 _levels did not differ from D4 to D12 within or between groups. RER and oxidation rates indicated that fat was the primary fuel source at rest; and, did not differ within or between groups from D4 to D12.

**Table 3:** Respiratory Exchange Ratios and Oxidation Rates

	BAL-MP		DEF-MP		DEF-HP	
	**Baseline (D1-4)**	**Intervention (D5-11)**	**Baseline (D1-4)**	**Intervention (D5-11)**	**Baseline (D1-4)**	**Intervention (D5-11)**

RER (VCO_2_/VO_2_)	0.76 ± 0.05	0.76 ± 0.04	0.73 ± 0.03	0.74 ± 0.01	0.75 ± 0.04	0.75 ± 0.03
VO_2 _(mL/min)	267 ± 18	273 ± 26	271 ± 26	268 ± 26	319 ± 36	306 ± 40
Carbohydrate Oxidation Rate (g/min)^1^	76 ± 58	59 ± 44	33 ± 14	46 ± 44	65 ± 51	62 ± 40
Fat Oxidation Rate (g/min)^2^	102 ± 30	112 ± 25	122 ± 12	115 ± 23	133 ± 25	127 ± 20

Values expressed as means ± SD; RER = respiratory exchange ratio; VCO_2 _= carbon dioxide production; VO_2 _= oxygen consumption)

^1^Calculated using the following equation: 4.21(VCO_2_) - 2.962(VO_2_)

^2^Calculated using the following equation: 1.695(VO_2_) - 1.701(VCO_2_)

### Glucose and Gluconeogenesis Kinetics

**Figure 2:**
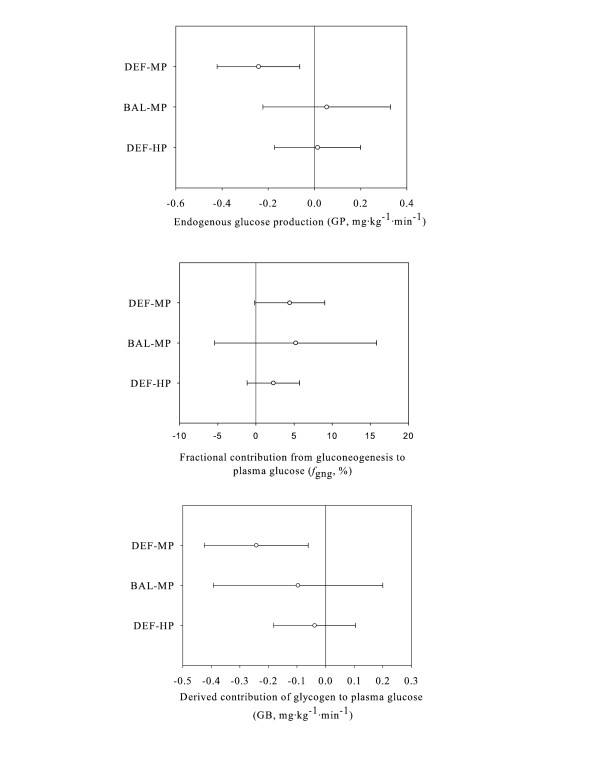
**Glucose and gluconeogenesis kinetics: mean change from D4 to D12 and 95% confidence intervals for each diet group**. This figure presents the mean change from D4 and 95% confidence intervals for each diet group for glucose production (GP), gluconeogenesis (*f*_gng_), and glycogen breakdown (GB). GP declined in response to the increase in exercise without any change in diet, but did not change with concomitant increase in dietary energy or protein. There were no changes in *f*_gng _and no significant differences between groups (p > 0.05). GB declined in response to the increase in exercise. Provision of additional dietary protein or energy appears to have attenuated the response. No significant differences between groups were observed for GP, *f*_gng_, or GB.

Comparison between groups with respect to time for GP, *f*_gng_, and GB are shown in Table 4, while Figure 2 represents the mean change from D4 and 95% confidence intervals for each diet group in terms of GP, *f*_gng_, and GB. A main diet effect and significant diet × time interaction was observed, whereby GP declined over time for DEF-MP, but not BAL-MP or DEF-HP, p < 0.05. The mean change and 95% confidence intervals demonstrates that GP declined in response to the increase in exercise without any change in diet, but did not change with concomitant increase in dietary energy or protein (Figure 2). Further, a tendency for a significant difference in the mean change in GP from D4 between DEF-MP and BAL-MP was noted (P = 0.06), with no significant differences observed between DEF-MP and DEF-HP.

**Table 4:** Glucose production, hepatic glucose flux, and blood chemistries: ANOVA results

	D4	D12	Diet × Time Interaction (p-value)
Endogenous Glucose Production (GP, g·kg^-1^·min^-1^)			

DEF-MP^a^	2.3 ± 0.3	2.1 ± 0.2	
BAL-MP^b^	2.7 ± 0.5	2.8 ± 0.4	0.046
DEF-HP^b^	2.3 ± 0.2	2.3 ± 0.3	

Contribution of Gluconeogenesis to Endogenous Glucose Production (*f*_*gng*_, %)			

DEF-MP	40.0 ± 6.0	44.4 ± 3.4	
BAL-MP	37.3 ± 7.1	42.6 ± 6.5	0.736
DEF-HP	44.6 ± 4.8	46.9 ± 4.5	

Absolute flux from glycogen to plasma glucose (GB, g·kg^-1^·min^-1^)			

DEF-MP	1.4 ± 0.3	1.2 ± 0.2	
BAL-MP	1.7 ± 0.2	1.6 ± 0.2	0.231
DEF-HP	1.3 ± 0.2	1.2 ± 0.2	

Glucose (mg·dL^-1^)			

DEF-MP	92.7 ± 10.8	88.1 ± 7.4	
BAL-MP	91.0 ± 7.6	89.2 ± 3.9	0.54
DEF-HP	92.5 ± 5.2	86.5 ± 4.2	

Glycerol (mmol·L^-1^)			

DEF-MP	0.04 ± 0.03	0.03 ± 0.02	
BAL-MP	0.06 ± 0.03	0.04 ± 0.03	0.19
DEF-HP	0.02 ± 0.02	0.04 ± 0.03	

Free Fatty Acids (mmol·L^-1^)			

DEF-MP	0.49 ± 0.16	0.55 ± 0.18	
BAL-MP	0.56 ± 0.29	0.46 ± 0.13	0.42
DEF-HP	0.51 ± 0.13	0.61 ± 0.24	

β-hydoxybutyrate (mmol·L^-1^)			

DEF-MP^a^	0.12 ± 0.78	0.25 ± 0.13	
BAL-MP^a^	0.15 ± 0.09	0.16 ± 0.09	0.02
DEF-HP^b^	0.13 ± 0.08	0.34 ± 0.22	

Insulin (uU·mL^-1^)			

DEF-MP	6.9 ± 2.9	4.3 ± 0.5	
BAL-MP	5.5 ± 2.1	4.2 ± 0.6	0.18
DEF-HP	8.2 ± 2.6	4.4 ± 2.3	

Glucagon (pg·mL^-1^)			

DEF-MP	42.3 ± 17.8	32.9 ± 13.9	
BAL-MP	42.1 ± 20.1	29.6 ± 6.9	0.89
DEF-HP	68.1 ± 17.6	54.9 ± 12.6	

Values expressed as means ± SD. Unlike superscripts indicate significant between group diet × time differences (p < 0.05) as determined from post-hoc analysis.

A significant main effect of time was observed, whereby *f*_gng _was higher on D12 (44.6 ± 4.9%) compared to D4 (40.6 ± 6.4%) regardless of group, p < 0.05. The mean change and 95% confidence intervals indicates no changes in *f*_gng _and no significant differences between groups (p < 0.05).

A significant main effect for diet and time indicates that GB was higher for BAL-MP (1.64 ± 0.20 mg·kg^-1^·min^-1^) compared to both DEF-MP (1.28 ± 0.19 mg·kg^-1^·min^-1^) and DEF-HP (1.24 ± 0.20 mg·kg^-1^·min^-1^) and declined over time (D4: 1.44 ± 0.28 mg·kg^-1^·min^-1^; D12: 1.31 ± 0.28 mg·kg^-1^·min^-1^), respectively, p < 0.05. The mean change and 95% confidence intervals demonstrates that GB declined in response to the increase in exercise; however, provision of additional dietary protein or energy appears to have attenuated the response as the mean change and 95% confidence intervals were not different from zero. Further, no significant differences between groups were observed.

### Blood Biochemistries

**Figure 3:**
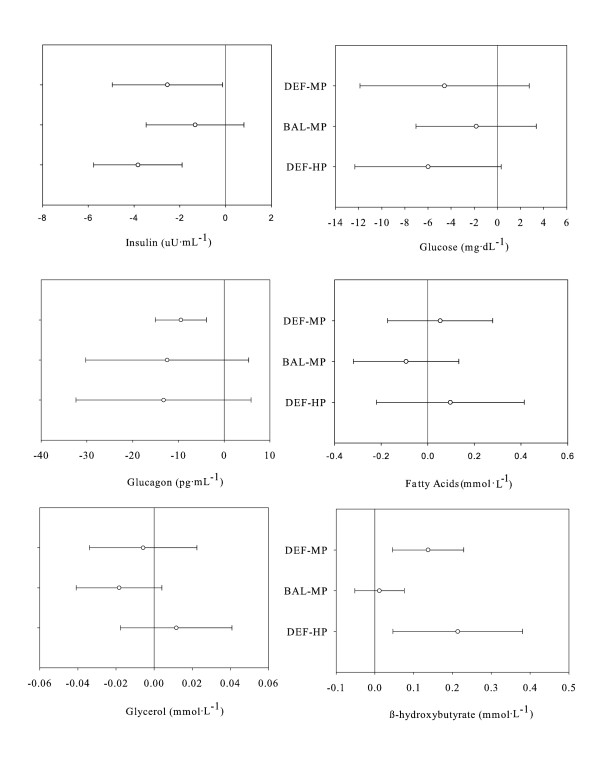
**Blood chemistries: mean change from D4 to D12 and 95% confidence intervals for each diet group**. This figure represents the mean change from D4 and 95% confidence intervals for each diet group in terms of insulin, glucose, glucagon, glycerol, free fatty acids, β-Hydroxybutyrate, glucagon, and insulin. Insulin declined in response to the increased exercise, however, increased energy appears to have attenuated the response. No changes were noted for glucose and there were no significant differences between groups for either glucose or insulin (p < 0.05). Glucagon declined in response to the increase in exercise. Provision of additional dietary protein or energy appears to have attenuated the response, although the change in glucagon was not statistically different between groups. No changes, and no significant between-group differences, were observed for free fatty acids or glycerol (p > 0.05). β-Hydroxybutyrate increased in response to the increased exercise, however, additional energy appears to have attenuated the response. The increase over time in β-Hydroxybutyrate for DEF-HP was greater compared to BAL-MP and DEF-MP (p < 0.05).

Comparison between groups with respect to time for glucose, glycerol, free fatty acids, β-Hydroxybutyrate, glucagon, and insulin are shown in Table 4, while Figure 3 represents the mean change from D4 and 95% confidence intervals for each diet group in terms of glucose, glycerol, free fatty acids, β-Hydroxybutyrate, glucagon, and insulin.

A significant main effect of time was observed, whereby β-Hydroxybutyrate was higher on D12 (0.25 ± 0.16 mmol·L^-1^) compared to D4 (0.13 ± 0.08 mmol·L^-1^) regardless of group, p < 0.05. The mean change and 95% confidence intervals indicated that β-Hydroxybutyrate increased in response to the increased exercise, however, additional energy appears to have attenuated the response as the mean change and 95% confidence intervals were not different from zero. Further, the increase over time for DEF-HP was greater compared to BAL-MP and DEF-MP (p < 0.05).

A significant main effect of time was observed, whereby glucose and insulin declined from D4 to D12 regardless of group (glucose: 92.1 ± 7.9 mg·dL^-1 ^and 88.0 ± 5.3 mg·dL^-1^, respectively; and, insulin: 6.9 ± 2.7 μU·mL^-1 ^and 4.3 ± 1.3 μU·mL^-1^, respectively, p < 0.05). The mean change and 95% confidence intervals indicates that insulin declined in response to the increased exercise, however, increased energy appears to have attenuated the response as the mean change and 95% confidence intervals were not different from zero. No changes were noted for glucose and there were no significant differences between groups for either glucose or insulin (p < 0.05).

A significant main effect for diet and time indicates that glucagon was higher for DEF-HP (61.5 ± 16.1 pg·mL^-1^) compared to both BAL-MP (35.8 ± 15.7 pg·mL^-1^) and DEF-MP (37.6 ± 16.1 pg·mL^-1^) and declined over time (D4: 50.4 ± 21.4 pg·mL^-1^; D12: 38.8 ± 15.8 pg·mL^-1^), respectively, p < 0.05. The mean change and 95% confidence intervals demonstrated that glucagon declined in response to the increase in exercise; however, provision of additional dietary protein or energy appears to have attenuated the response as the mean change and 95% confidence intervals were not different from zero. Further, no significant differences between groups were observed.

There were no significant main effects for diet or time in terms of NEFA or glycerol. Further, the mean change and 95% confidence intervals indicate no changes in NEFA or glycerol and no significant differences between groups (p > 0.05).

## Discussion

In the present investigation, we sought to determine if the rate of endogenous glucose production declines in response to an abrupt increase in energy expenditure and to ascertain if dietary manipulation- provision of additional dietary protein with concomitant negative energy balance-would attenuate this effect. The primary observations were that a 1000 kcal·d^-1 ^exercise-induced energy deficit caused a decline in GP attributable to a decline in GB, but provision of additional dietary protein mitigated the effects of a sudden increase in exercise on GP. We observed no difference within and between groups in terms of resting RER and oxidation rates. However, we did observe an increase in β-Hydroxybutyrate, and decrease in glucose and insulin, in response to the unaccustomed increase in energy expenditure regardless of dietary protein or energy intake.

As expected, RERs at baseline for all groups were approximately 0.75, indicating that fat was the main substrate oxidized at rest (i.e., ~84% of energy at rest was derived from fat). This is consistent with other reports in aerobically-trained subjects [20]. Lack of within or between group changes over time in RER and oxidation rates was not surprising, given that subjects were already predominantly oxidizing fat at rest prior to the energy deficit phase. It does contrast, however, with the observation that endogenous carbohydrate oxidation is reduced at rest during periods of energy deficit [21].

The fact that volunteers receiving a higher protein diet were able to maintain GP during the period of increased energy expenditure and negative energy balance suggests that manipulation of macronutrient intake partially offsets the energy deficit-associated fall in glucose production when an increase in exercise to create an energy deficit is undertaken for weight loss or when negative energy balance is inevitable (for example, in the case of military operations or prolonged exercise). The most likely mechanism to explain this effect relates to our observation that maintenance of GB seems to have attenuated the decline in GP associated with the exercise-induced energy deficit. Since the current study did not have adequate power to detect a difference in GB between DEF-HP and DEF-MP using 2-way ANOVA, more rigorous studies are needed to further investigate this speculation. Future studies, aiming to detect a 0.4 g·kg^-1^·min^-1 ^difference in GB between groups (standard deviation 0.2 g·kg^-1^·min^-1^; α = 0.05; power = 0.80), should test at least eight volunteers per group.

The ability of DEF-HP to maintain GP may reflect the increased availability of gluconeogenic substrates. We detected no between-group differences in *f*_gng _and free fatty acids, during the period of insufficient energy intake. However, β-Hydroxybutyrate, which stimulates gluconeogenesis, increased in response to the increased exercise, particularly in the two energy deficit groups. More importantly, the increase over time in β-Hydroxybutyrate for DEF-HP was significantly higher compared to BAL-MP and DEF-MP. Increased reliance on alanine-derived gluconeogenesis is another possibility, since the glucose-alanine cycle is up-regulated during exercise and the rate of glucose production from alanine is even higher during periods of starvation [22], although we have no data to support this hypothesis. Lastly, an increase in the recycling of glucose via fat stores may occur, since periods of starvation stimulate the mobilization of fat [23] and total glucose production from glycerol may be ≥ 20% during starvation [24]. Our results, however, do not support this mechanism, since we observed no differences in fatty acid availability in response to the exercise-induced energy deficit. Further, the lypolytic response appears to differ between an exercise-induced energy deficit and a diet-induced energy deficit.

The observation that GP declined in response to an exercise-induced energy deficit is consistent with studies investigating negative energy balance due to restricted energy intake. Indeed, a decline in glucose production has been observed by Hellerstein et al. [11] in response to prolonged starvation (60 hours), likely due to reduced availability of alanine and lactate to the liver [25]. This finding is also consistent with our observation that insulin levels declined in response to the exercise-induced energy deficit. We also observed maintenance of GP and insulin levels when energy balance was preserved despite increased exercise and total energy expenditure. This was also predictable, given that endogenous glucose production is maintained during periods of energy balance [11].

The observation that the high protein group was capable of preserving GP is suggestive that dietary protein supplementation may not only help with preservation of lean mass during periods of energy restriction [8-10], but may also have utility for preserving metabolic flexibility. These findings may also have implications for overweight individuals who purposely induce an energy deficit via an increase in exercise to achieve weight loss; however, future research is needed to confirm this hypothesis.

Regardless of energy or protein intake, *f*_gng _appears to have increased while GB appears to have decreased in response to an increase in energy expenditure (Table 3) although analysis of the confidence intervals is not entirely supportive (Figure 2). The pattern for increased *f*_gng _observed in this study is consistent with Bergman et al. [26], who reported an increase in gluconeogenesis in response to nine weeks of unaccustomed endurance training. Our findings are not surprising, given that glycogen stores likely decline over time in response to the demands of an unaccustomed increase in exercise thus placing greater reliance on *f*_gng _to maintain GP. This hepatic auto-regulation has been demonstrated consistently in the literature, for example, when glycogen stores are reduced in response to prolonged starvation, *f*_gng _increases in an attempt to maintain GP [11]. Further, Staehr et al. [13] reported that when galactose was given to stimulate GB, gluconeogenesis declined in a compensatory manner and GP was maintained. The current study provides further evidence of hepatic auto-regulation in terms of glucose metabolism.

Although it is plausible that depleted glycogen stores may have been the primary factor in the apparent reduction in GB, lower plasma glucagon concentrations may also have played a role. Indeed, the DEF-MP group experienced a significant decrease in fasting glucagon in response to increased energy expenditure. This result is consistent with the findings of Tremblay et al. [27] and Oppert et al. [28] who found that fasting glucagon levels tended to decrease following 100 days of a 1000 kcal/d exercise-induced energy deficit. Further, hepatic glucagon sensitivity increases in response to unaccustomed endurance exercise [29], such that less glucagon is needed to evoke a given level of GP; and, fasting may elicit a similar response [30]. Moreover, the maintenance of plasma glucagon levels over time for the DEF-HP group may be attributed to the higher protein content of the diet. Indeed, fasting plasma glucagon has been shown to be higher in energy-balanced individuals who habitually consume high-compared to normal-protein diets (~1.9 g/kg/d and ~0.75 g/kg/d, respectively, for 6 months), which was accompanied by a concomitant rise in gluconeogenesis in the high protein group [31].

Despite the differential effects of diet on GP, we did not observe differential effects on plasma glucose over time. Statistical analysis of the glucose values on day 4 and day 12 indicated that glucose fell over time; independent of group. However, the change-over-time scores visually suggest that the high protein group trend towards a greater decline over time. With that being said, the magnitude of the change over time is small. Moreover, the statistical tests were inconsistent with 2-way repeated measures ANOVA indicating a change across time; whereas the 95% confidence intervals for change scores of the diet groups overlap with zero change, suggesting that the real response may or may not have been different from the null. Our conclusion is that the glucose data should not be over-interpreted.

Generalizing study results to females should be done with caution, since the current study included males-only. Additionally, the methodology used to measure *f*_gng _should be taken into consideration when interpreting this data. The contribution of gluconeogenesis to endogenous glucose production observed in this study at baseline (i.e. prior to the increase in exercise energy expenditure) is similar to other studies employing the MIDA technique to estimate gluconeogenic flux [11,32]. Differences between MIDA and other methods for estimating *f*_gng _have been presented in the literature [33,34]. Briefly, *f*_gng _values determined via MIDA tend to be lower compared to nuclear magnetic resonance and liver biopsy methods, since MIDA involves the calculation of GB from measurements of GP and *f*_gng _whereas the other methods ascertain *f*_gng _via the difference between the measured GP and net change in glycogen [32]. Nevertheless, the present investigation observed an increase in *f*_gng _in response to the increase in energy expenditure, which appears to be an adaptation to the unaccustomed increase in exercise.

## Conclusions

The current study demonstrates that energy balance is ideal for maintaining glucose production during a sudden increase in energy expenditure. However, when provision of energy is not sufficient to meet the demands of an increase in energy expenditure or in individuals trying to lose weight via an increase in energy expenditure, increasing the protein content may be a viable alternative for maintaining glucose production. Future studies should investigate whether the maintenance of glucose production at rest is beneficial during exercise in terms of fuel utilization and performance indices.

## Competing interests

The authors declare that they have no competing interests.

## Authors' contributions

TS coordinated data acquisition, performed the statistical analysis, and drafted the manuscript. JMS and JR were responsible for analysis of glucose and glycerol kinetics. SM assisted with statistical analysis, and critically revised the manuscript for intellectual content. MP participated in data acquisition and analysis, and helped to draft the manuscript. EG participated in data acquisition and analysis related to the exercise intervention. CC participated in study design and was responsible for data acquisition related to glucose and glycerol kinetics. AY made substantial contributions to study conception and design and critically revised the manuscript for intellectual content. All authors read and approved the final manuscript.
